# Kidney replacement and conservative therapies in rhabdomyolysis: a retrospective analysis

**DOI:** 10.1186/s12882-024-03536-8

**Published:** 2024-03-14

**Authors:** Jonathan de Fallois, Robert Scharm, Tom H. Lindner, Christina Scharf, Sirak Petros, Lorenz Weidhase

**Affiliations:** 1 Medical Department III, Division of Nephrology, University of Leipzig Medical Center, Leipzig, Germany; 2 Medical Intensive Care Unit, Medical ICU, University of Leipzig Medical Center, University Hospital of Leipzig, Liebigstr. 20, 04103 Leipzig, Germany; 3 Department of Anesthesiology, University Hospital, LMU Munich, Munich, Germany

**Keywords:** Rhabdomyolysis, Kidney replacement therapy, Myoglobin elimination, Akute kidney injury

## Abstract

**Background:**

Toxic renal effects of myoglobin following rhabdomyolysis can cause acute kidney injury (AKI) with the necessity of kidney replacement therapy (KRT). Fast elimination of myoglobin seems notable to save kidney function and intensify kidney repair. Clinical data regarding efficacy of KRT in critical care patients with rhabdomyolysis and AKI are limited. This retrospective analysis aimed to identify differences between conservative therapy and different modalities of KRT regarding myoglobin elimination and clinical outcome.

**Methods:**

This systematic, retrospective, single-center study analyzed 328 critical care patients with rhabdomyolysis (myoglobin > 1000 µg/l). Median reduction rate of myoglobin after starting KRT was calculated and compared for different modalities. Multivariate logistic regression models were established to identify potential confounder on hospital mortality. Filter lifetime of the various extracorporeal circuits was analyzed by Kaplan–Meier curves.

**Results:**

From 328 included patients 171 required KRT. Health condition at admission of this group was more critical compared to patient with conservative therapy. Myoglobin reduction rate did not differ between the groups (KRT 49% [30.8%; 72.2%] vs. conservative treatment (CT) 61% [38.5%; 73.5%]; *p* = 0.082). Comparison between various extracorporeal procedures concerning mortality showed no significant differences. Hospital mortality was 55.6% among patients with KRT and 18.5% with CT (*p* < 0.001). Multivariate logistic regression model identified requirement for KRT (OR: 2.163; CI: 1.061–4.407); *p* = 0.034) and the SOFA Score (OR: 1.111; CI: 1.004–1.228; *p* = 0.041) as independent predictive factors for hospital mortality. When comparing specific KRT using multivariate regression, no benefit was demonstrated for any treatment modality. Life span of the extracorporeal circuit was shorter with CVVH compared to that of others (log-Rank *p* = 0.017).

**Conclusions:**

This study emphasizes that AKI requiring KRT following rhabdomyolysis is accompanied by high mortality rate. Differences in myoglobin reduction rate between various KRTs could not be confirmed, but CVVH was associated with reduced filter lifetime compared to other KRTs, which enable myoglobin elimination, too.

**Graphical Abstract:**

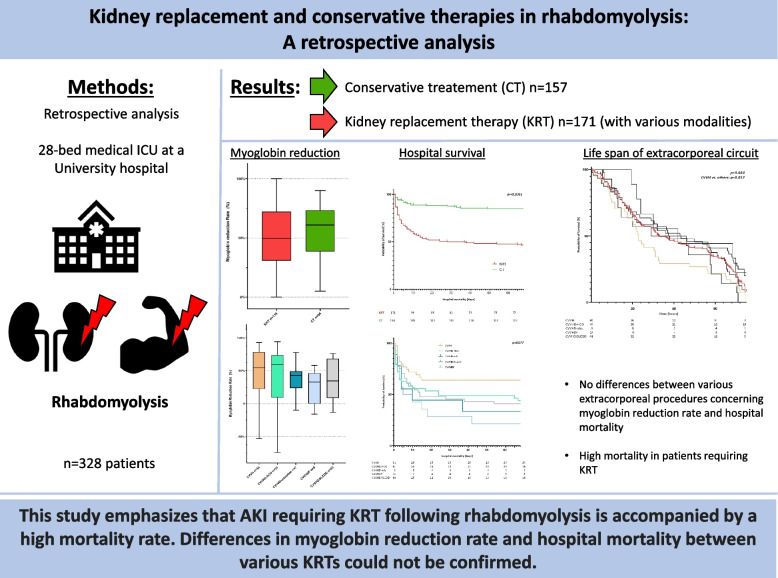

**Supplementary Information:**

The online version contains supplementary material available at 10.1186/s12882-024-03536-8.

## Background

Acute kidney injury (AKI) is one of the leading organ dysfunctions in critically ill patients and is associated with high mortality and morbidity rates [1–3], high economic costs to health care systems [4], and increases the risk for chronic kidney disease (CKD) [5]. Different etiologies are involved in the development of AKI in most critically ill patients, whereby the frequent reasons are septic or cardiogenic shock, post-major surgery, hypovolemia or drug induced toxicity [3]. Rhabdomyolysis is considered as the potential cause of approximately 5–25% [6, 7].

Damaged skeletal muscle releases muscle cell contents into the circulation, e.g. myoglobin and other molecules. Myoglobin is endocytosed and oxidized by tubular cells, resulting in radical oxygen species that alter DNA and proteins. It activates an inflammatory response in the kidney and mediates vasoconstriction, which perpetuates renal damage. Myoglobin is also filtered by the glomerulus and precipitates in the renal tubules, particularly in combination with the Tamm–Horsfall proteins, forming tubular casts, which consequently result in acute tubular obstruction [6, 8].

Fast elimination of myoglobin seems essential due to its direct toxic renal effects [8]. However, clinical data regarding the efficacy of different kidney replacement therapy (KRT) modalities are limited. With normal diffusion-based hemodialysis it is usually not possible to effectively eliminate molecules with middle molecular weight, such as myoglobin (17 kDa). In critical care patients this is possible through convective transport such as continuous venovenous hemofiltration (CVVH) [9–11] or as well continuous veno-venous hemodiafiltration (CVVHDF) [12, 13].

Myoglobin can also be eliminated by diffusive transport by using high-cut-off filters (HCO) with a pore size larger than 0.01 μm in continuous venovenous hemodialysis (CVVHD-HCO) [12, 14–17]. The third way to eliminate myoglobin with KRT is to integrate an adsorber into the extracorporeal circuit [18–21].

Due to the paucity of clinical data that compare modalities of KRT despite the high prevalence of rhabdomyolysis induced AKI, we retrospectively analyzed patients with AKI and rhabdomyolysis admitted to our medical intensive care unit (ICU) from January 2016 to August 2020. Primary outcome measure was to identify influencing factors on the in-hospital mortality in patients with severe rhabdomyolysis. Secondary outcome measure was the evaluation of different dialysis modalities in consideration of their effectiveness.

## Methods

### Study design

This systematic retrospective, single-center study was approved by the local Institutional Review Board at the Medical Faculty of the University of Leipzig (312/20-ek).

To address the question whether reduction in serum myoglobin concentrations by extracorporeal circuit might influence patient outcome, we planned to compare the course of myoglobin reduction rate (MRR) between different treatment modalities. Furthermore, we aimed to investigate other possible effects on clinical and kidney outcome among these patients.

### Patients

**Fig. 1:**
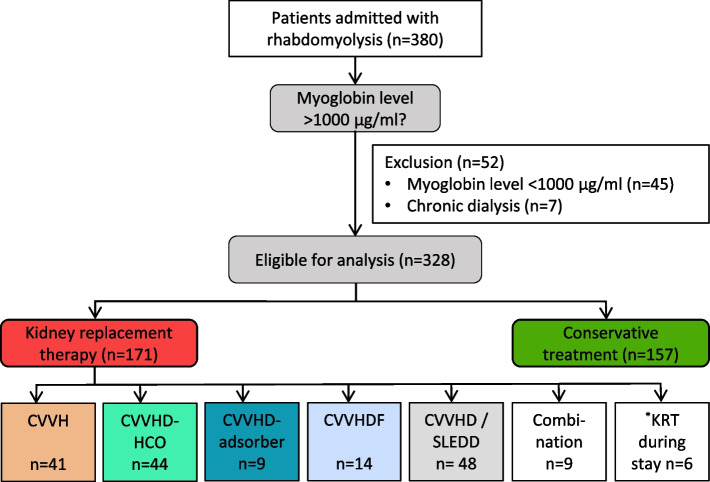
Study flow chart. *KRT during stay: KRT not at the beginning of the ICU stay, but later; CVVH continuous veno-venous hemofiltration, CVVHD-HCO continuous veno-venous hemodialysis with high cut-off filter, CVVHD-adsorber continuous veno-venous hemodialysis with “standard” high-flux filter and adsorber, CVVHDF continuous veno-venous hemodiafiltration, SLEDD sustained low efficiency daily dialysis

A total of 380 patients with the diagnosis of rhabdomyolysis were treated in our 28-bed medical ICU at the University Hospital Leipzig during the study period. After reviewing the medical records, we excluded 45 patients from further analysis because they did not experience severe rhabdomyolysis and seven because of preexisting dialysis dependency. Based on thresholds from published studies, rhabdomyolysis was defined as a serum myoglobin level > 1000 µg/l [22, 23]. Patients were enrolled to the study at the point of the first measured myoglobin level > 1000 µg/L following ICU admission. In the final analysis, 328 patients were considered for further evaluation (Fig. 1).

Firstly, we grouped these patients based on the management received to either KRT or conservative treatment group (CT). In a second step, we planned to describe the patients with KRT more precisely.

### Procedures

Conservative therapy was the preferred therapeutic intervention, based on available recommendations from literature. Initially, targeted parenteral fluid therapy was carried out to compensate for a possible volume deficit and to strictly control the fluid balance [24]. Secondly, urine pH was controlled and if necessary alkalized by citrate-containing drugs three times a day. Success was monitored by urine output and urine pH with a target above 6.8 [25, 26]. Decision to start KRT and the choice of treatment modality were made by the attending intensivists according the current recommendations [27, 28].

During the observation period, patients with AKI and rhabdomyolysis received treatment with continuous kidney replacement therapy (CVVH, CVVHD; CVVHDF) with multiFiltrate® device and sustained low-efficiency daily dialysis (SLEDD; GENIUS®90) (both Fresenius Medical Care, Bad Homburg, Germany). A postdilution CVVH was applied with Ultraflux AV 600S® and CVVHDF with Ultraflux AV1000S® filter. In case of CVVHD-HCO, Ultraflux EMiC2® was applied (all Fresenius Medical Care, Bad Homburg, Germany). An adsorber (CytoSorb®, CytoSorbents Europe GmbH, Müggelseedamm 131, Berlin, Germany) was sequentially inserted into the circuit while using CVVHD with high-flux filter Ultraflux AV1000S®). SLEDD was performed with GENIUS® sleddFlux. All filters consisted of poly-sulfone with a pore size of approximately 30 kilodalton (kDa) for the high-flux filters and 45 kDa for the high cut-off filters. Anticoagulation of the extracorporeal circuit was maintained by regional citrate anticoagulation (RCA), whenever possible. Systemic anticoagulation was only performed in patients with other medical reasons justifying therapeutic anticoagulation, citrate intolerance or patients using CVVH. Hemofiltration requires a higher blood flow of the extracorporeal circuit than diffusion-based techniques due to the hemoconcentration at the filter. Necessity of higher citrate intake increases the risk of citrate accumulation in patients with severe organ dysfunction. For this reason, in our medical ICU, CVVH with RCA was not performed. Total turnover rate (TTR, dialysate and replacement fluid) in cases treated with continuous KRT was adjusted at about 25 ml/kg ideal or adjusted body weight/h [29]. SLEDD ran according to clinical requirements, mostly every 2 days.

### Data collection

The following clinical characteristics and demographic data were retrieved from electronic health records of the patients at the time of ICU admission: age, sex, height, weight, BMI, kidney function and AKI stage, admission diagnosis, indication for KRT, acute physiology and chronic health evaluation II (APACHE II) and sequential organ failure assessment (SOFA) score, mean arterial blood pressure, need for mechanical ventilation, need for vasopressor, sepsis, pre-existing diseases and cause for rhabdomyolysis.

These data were collected daily during the observation period: creatinine, urea, albumin, creatine kinase (CK), myoglobin, interleukin-6 (IL-6), pH, bicarbonate, potassium, calcium, phosphate, lactate, platelet- and white blood cell count, urine output, urine pH. Duration of KRT and filter lifetime of the extracorporeal circuit were recorded.

Clinical follow-up data were monitored and retrieved from the last documented hospital or outpatient contact (length of ICU stay (days), kidney function and all-cause mortality at hospital discharge and, if available, at day 90).

### Endpoints and calculations

For comparison of the different therapeutic interventions, we calculated MRR after starting therapeutic intervention or extracorporeal circuit, respectively.

$$1-\left(\frac{C_{myoglobin\;T2}}{C_{myoglobin\;T1}}\right)$$

In most cases, myoglobin was daily measured, so we calculated MRR between the first (C_myoglobin T1_) and the second morning (C_myoglobin T2_) after ICU admission.

### Statistical analysis

Categorical variables are displayed as frequencies and percentages and tested by Chi-square (two-sided) test. Continuous variables are given as median with 25th and 75th quartile in square brackets based on test for normal distribution using the Shapiro–Wilk test. Normally distributed variables were analyzed by Student’s t-test and not normally distributed variables by Mann–Whitney U test.

The impact of different parameters on all-cause hospital mortality for the total cohort was tested by means of a logistic regression model. The following variables were included in this model: age, sex, height, weight, BMI and preexisting comorbidities (immunosuppressive therapy, CKD > stage 3a before admission, active malignancy, chronic heart failure, liver cirrhosis, chronic pulmonary disease) and the health condition at admission (AKI stage, need for KRT, non-invasive/invasive mechanical ventilation, vasopressors, sepsis/septic shock, MAP, heart rate, diuresis, urine pH, APACHE II/SOFA score and urea, creatinine, pH, HCO_3_^−^, potassium, lactate, IL-6, platelets, leukocytes, albumin, phosphate, ionized calcium, CK and myoglobin levels). This multivariate calculation had been only carried out in cases with complete data sets and for significant variables after univariate testing. Kaplan–Meier survival curves were used to depict hospital mortality and applied to the performance of extracorporeal circuits.

Additionally, the influence of the aforementioned variables and the different KRT modalities (CVVH, CVVHD-HCO, CVVHD-adsorber, CVVHDF, CVVHD/SLEDD) on hospital mortality was also tested for the KRT cohort using a logistic regression model. In this case, the specific KRT modality was tested in comparison to all other KRT modalities. Missing data and loss of follow up are addressed in detail.

Statistical analysis was performed using IBM SPSS, versions 27 and GraphPad Prism version 9. The significance level was defined as 5% for two-tailed tests.

## Results

### Patient characteristics

Demographic and clinical characteristics of the patients are given in Table 1. Patients on KRT were more seriously ill than those with no need for KRT illustrated by higher APACHE II and SOFA scores. Preexisting chronic heart failure (NYHA IV), preexisting immunosuppressive therapy, liver cirrhosis, chronic pulmonary disease or chronic kidney disease with eGFR below 45 ml/kg/1.72m^2^ did not differ between both groups. Nevertheless, active malignancy was associated with a higher percentage of need for KRT. Patients with KRT had more rhabdomyolysis triggered by shock or after cardio-pulmonary resuscitation. However, muscle hypoxia was slightly more causal in patients with CT, including crush syndrome and limb ischemia. Other triggers of rhabdomyolysis were similar.

**Table 1:** Patient characteristics

Parameter	All (*n* = 328)	KRT (*n* = 171)	CT (*n* = 157)	*p*
Age (years)	69 [56; 79]	69 [55; 76]	69 [56; 81]	0.098
Sex (male) n, (%)	218 (66.5)	117 (68.4)	101 (64.3)	0.433
High (cm)	173 [166; 180]	174 [165; 180]	173 [167; 178]	0.584
Weight (kg)	80 [69; 92]	80 [70; 94]	77 [68; 90]	0.086
BMI (kg/m^2^)	26.23 [23.2; 30.0]	26.6 [23.2; 30.7]	25.5 [23.2; 28.7]	0.143
Mechanical ventilation n, (%)	116 (35.4)	82 (48.0)	34 (21.7)	**< 0.001**
Sepsis n, (%)	112 (34.1)	77 (45.0)	35 (22.3)	**< 0.001**
Vasopressors n, (%)	135 (41.2)	101 (59.1)	34 (21.7)	**< 0.001**
AKI 3 n, (%)	119 (36.3)	97 (56.7)	22 (14.0)	**< 0.001**
SOFA n, (%)	7 [3; 12]	11 [7; 14]	4 [2; 7]	**< 0.001**
APACHE II	27 [19; 33]	32 [26; 38]	21 [16; 28]	**< 0.001**
MAP (mmHg)	86 [69; 103]	81 [63; 96]	94 [75; 110]	**< 0.001**
HR (bpm)	96 [81; 111]	101 [84; 119]	89 [79; 105]	**< 0.001**
**Preexisting condition:**				
Chronic heart failure (NYHA IV) n, (%)	5 (1.5)	3 (1.8)	2 (1.3)	0.723
Pre-existing immunosuppression n, (%)	19 (5.8)	13 (7.6)	6 (3.8)	0.143
Liver cirrhosis n, (%)	20 (6.1)	12 (7.0)	8 (5.1)	0.467
Active malignancy n, (%)	17 (5.2)	14 (8.2)	3 (1.9)	**0.010**
Chronic pulmonary disease n, (%)	44 (13.4)	23 (13.5)	21 (13.4)	0.984
^a^eGFR < 45 ml/min/1.73m^2^ n, (%)	44/79^a^ (55.7)	24/38^a^ (63.2)	20/41^a^ (48.8)	0.199
**Causes of rhabdomyolysis:**				
Infection n, (%)	43 (13.1)	28 (16.4)	15 (9.6)	0.068
Muscle hypoxia n, (%)	176 (53.7)	78 (45.6)	98 (62.4)	**0.002**
Drugs and toxins n, (%)	16 (4.9)	9 (5.3)	7 (4.5)	0.735
Shock and post CPR n, (%)	34 (10.4)	27 (15.8)	7 (4.5)	**< 0.001**
Metabolic/electrolyte disorders n, (%)	3 (0.9)	0	3 (1.9)	0.069
Exertion n, (%)	5 (1.5)	3 (1.8)	2 (1.3)	0.723
Electrical current n, (%)	19 (5.8)	10 (5.8)	9 (5.7)	0.964
Other n, (%)	32 (9.8)	16 (9.4)	16 (10.2)	0.806

*AKI* Acute kidney injury, *APACHE* Acute Physiology And Chronic Health Evaluation, *BMI* Body mass index, *CPR* Cardiopulmonary resuscitation, *CT* Conservative treatment, *HR* Heart rate, *KRT* Kidney replacement therapy, *MAP* mean arterial pressure, *NYHA* New York Heart Association, *SOFA* Sequential organ failure assessment

^a^patients with data regarding kidney function before ICU admission

From 328 patients with myoglobin levels of > 1000 ug/l 54 (16.5%) patients had no AKI, 155 (47.3%) mild-to-moderate AKI (AKI 1 and 2) and 119 (36.3%) severe AKI (AKI 3). KRT was performed in 171 (52.1%) patients. Indication to start KRT had been pH < 7.2 (46/171; 26.9%), bicarbonate level < 12 mmol/l (22/171; 12.9%), potassium level > 6 mmol/l (28/171; 16.4%) and pulmonary edema (14/171; 8.2%). The remaining patients (61/171; 35.7%) presented with multiple reasons for starting KRT, such as prolonged oligoanuria, uremic syndrome or others. The KRT modalities provided are shown in Fig. 1. Dialytic parameters like total turnover rate and blood flow are listed in the Suppl. Table 1. Kidney function at baseline was more impaired in the KRT group, illustrated by higher creatinine, higher potassium levels, lower HCO_3_^−^ level, more acidic urine and less diuresis during the first 24 h after admission (Table 2).

**Table 2:** Laboratory parameters at diagnosis

Parameter	All (*n* = 328)	KRT (*n* = 171)	CT (*n* = 157)	*P*
Myoglobin (µg/l)	3441 [1664; 7462]	4855 [2169; 12385]	2459 [1532; 5576]	**< 0.001**
CK (µkat/l)	30.20 [14.7; 71.2]	30.36 [11.4; 72.7]	29.87 [17.6; 68.2]	0.722
Urea (mmol/l)	11.95 [7.3; 21.5]	13.5 [7.8; 24.8]	11.3 [6.8; 18.7]	**0.017**
Creatinine (µmol/l)	157.5 [107.0; 260.5]	200.0 [129.0; 313.0]	123.0 [90.5; 198.5]	**< 0.001**
IL-6 (pg/ml)	337 [121; 2246]	525 [198; 4412]	125 [72; 693]	**< 0.001**
Albumin (g/l)	29.80 [23.8; 36]	28.5 [22.5; 33.5]	33.9 [28.3; 40.5]	**< 0.001**
Phosphate (mmol/l)	1.45 [1.1; 2.1]	1.6 [1.1; 2.2]	1.3 [1; 1.61]	0.109
Ca^2+^ (mmol/l)	1.14 [1.1; 1.2]	1.12 [1.1; 1.2]	1.2 [1.1; 1.2]	**0.003**
Potassium (mmol/l)	4.40 [3.9; 5.1]	4.6 [4; 5.4]	4.2 [3.8; 4.8]	**< 0.001**
pH	7.34 [7.3; 7.4]	7.2 [7.2; 7.4]	7.4 [7.3; 7.4]	**< 0.001**
HCO_3_^−^ (mmol/l)	21.3 (17; 24.9]	19.3 [13.9; 23.2]	23.1 [19.5; 25.8]	**< 0.001**
Lactate (mmol/l)	2.6 [1.5; 5.1]	3.4 [1.9; 7.5]	1.9 [1.2; 3.5]	**< 0.001**
Platelets /µl	198 [130; 276]	182 [113; 266]	226 [150; 278]	**0.007**
Leucocytes/µl	14.5 [10.5; 19.6]	15.4 [9.9; 22]	13.7 [10.8; 17]	0.191
Urine pH	5.6 [5.0; 6.09]	5.0 [5.0; 6.0]	5.9 [5.0; 6.0]	**< 0.001**
24 h-diuresis (ml)	700 [250; 12289]	400 [100; 867]	1100 [500; 1655]	**< 0.001**

*CK* Creatine kinase, *CT* Conservative treatment, *IL-6* Interleukin 6, *KRT* Kidney replacement therapy

### Myoglobin reduction rate

**Fig. 2:**
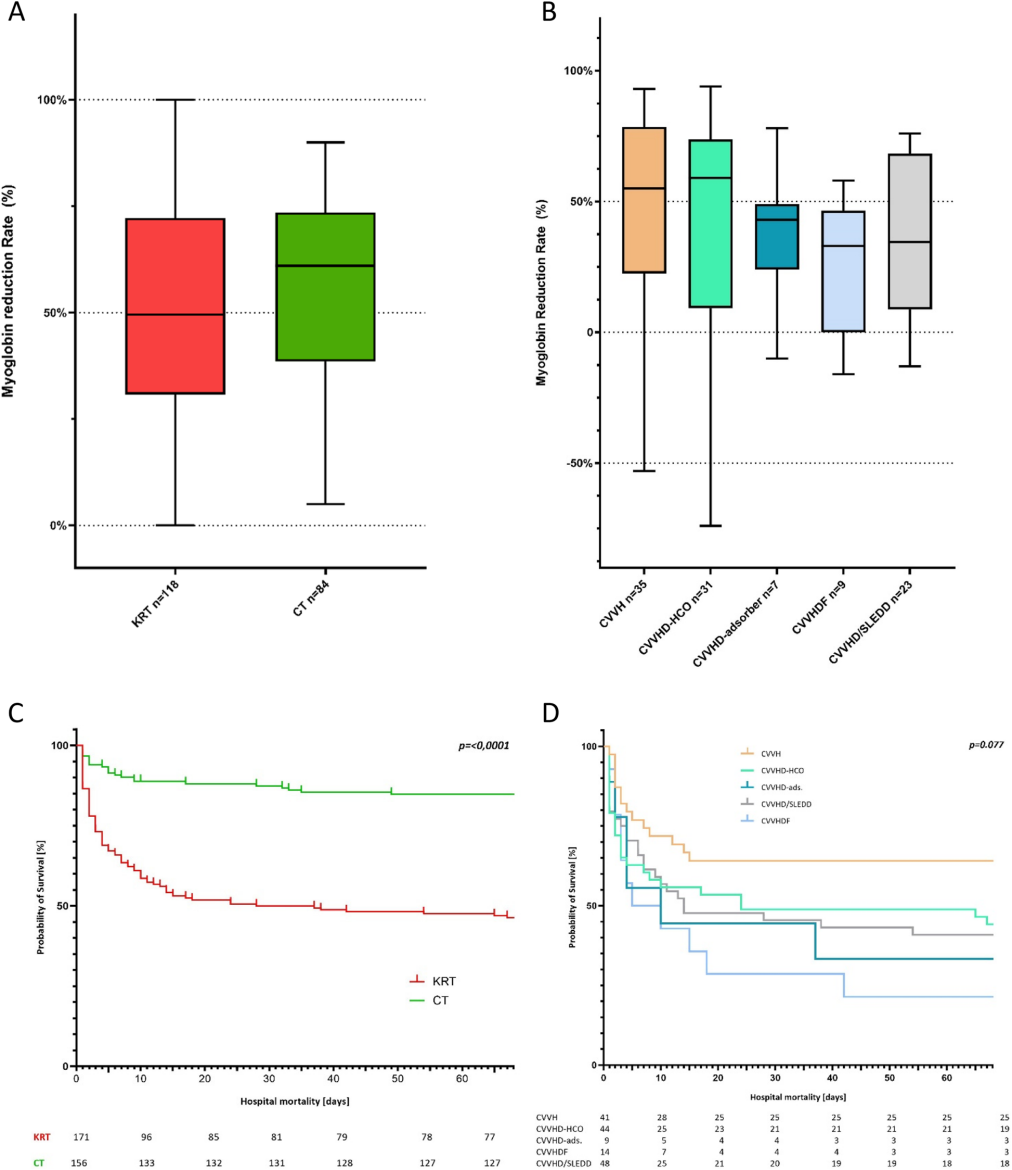
Myoglobin reduction rate. **A** Myoglobin reduction rates for kidney replacement therapy versus conservative treatment. KRT kidney replacement therapy, CT conservative treatment; **B** Myoglobin reduction rates for the various kidney replacement modalities. CVVH continuous veno-venous hemofiltration, CVVHD-HCO continuous veno-venous hemodialysis with high cut-off filter, CVVHD-adsorber continuous veno-venous hemodialysis with “standard” high-flux filter and adsorber, CVVHDF continuous veno-venous hemodiafiltration, CVVHD continuous veno-venous hemodialysis, SLEDD sustained low efficiency daily dialysis; **C** Kaplan–Meier curve for hospital survival for kidney replacement therapy versus conservative treatment. KRT kidney replacement therapy, CT conservative treatment; **D** Kaplan–Meier curve for hospital survival with the various kidney replacement modalities. CVVH continuous veno-venous hemofiltration, CVVHD-HCO continuous veno-venous hemodialysis with high cut-off filter, CVVHD-adsorber continuous veno-venous hemodialysis with “standard” high-flux filter and adsorber, CVVHDF continuous veno-venous hemodiafiltration, CVVHD continuous veno-venous hemodialysis, SLEDD sustained low efficiency daily dialysis

Both groups showed decreasing myoglobin levels regarding MRR between the first morning after enrollment and after 24 h. For the calculation of MRR, data on myoglobin level with a 24-h period were available in 61.1% (KRT: 69% and CT: 53.5%) of the included cases (Suppl. Table 2). The MRR was 49% [30.8%; 72.2%] with KRT and 61% [38.5%; 73.5%] with CT and showed no difference between the two groups (*p* = 0.082) (Fig. 2A). Despite therapeutic interventions and a myoglobin reduction in the overall group, some patients showed a continued increase in myoglobin levels (KRT: 24/118 (20.3%); CT: 11/84 (13.1%)). The comparison between the various extracorporeal procedures did not demonstrate a significant advantage regarding myoglobin elimination (Table 3 and Fig. 2B).

**Table 3:** Specific myoglobin reduction rate

	**All** ***n* = 118**	**CVVH** ***n* = 35**	**CVVHD-HCO** ***n* = 31**	**CVVHD-ads** ***n* = 7**	**CVVHDF** ***n* = 9**	**CVVHD/SLEDD** ***n* = 23**	**Others**^a^ ***n* = 13**
**MRR (%)**	49.5 [30.8; 72.3]	64 [38; 81]	61 [26.5; 74.3]	45 [35.3; 56.3]	33 [00; 48]	42 [20; 69]	
**P**^#^		0.7	0.266	0.787	0.161	0.708	

*ads.* Adsorber, *CVVH* Continuous veno-venous hemofiltration, *CVVHD* Continuous veno-venous hemodialysis, *CVVHDF* Continuous veno-venous hemodiafiltration, *HCO* High cut-off, *MRR* Myoglobin reduction rate, *SLEDD* Sustained low-efficiency daily dialysis

^#^Specific extracorporeal procedure compared with all other KRT modalities

^a^Others include KRT later during ICU stay and combination of more than one specific KRT: data not shown

### Clinical outcome

The ICU length of stay was 7 [2; 17] days in the KRT group, while it was 3 [2; 6] days for the CT group (*p* < 0.001). All-cause hospital mortality was 55.6% (95/171) in the KRT group and 18.5% (29/157) in the CT group (*p* < 0.001). Kaplan–Meier analysis illustrates Fig. 2C. Patients who died during hospital stay had significantly higher serum myoglobin levels on admission than survivors (4478 µg/ml [1824; 9794] vs. 2823 µg/ml [1590; 7107]; *p* = 0.036). However, admission myoglobin level was not an independent risk factor for hospital mortality in the logistic regression model. Data on kidney function on day 90 were available for only 42.6% (87/204) of survivors. The survivors did not differ in dialysis dependence (KRT: 2/33 (6.1%) versus CT: 1/54 (1.9%)) or having advanced kidney disease (eGFR < 45 ml/min/1.72m^2^: KRT 7/33 (21.2%) and CT 10/54 (18.5%); *p* = 0.76)). The eGFR was similar in both groups (KRT: 68 ml/min [51; 91], CT: 76 ml/min [50; 106]; *p* = 0.62). Serum myoglobin level on ICU admission was not predictive for kidney function on day 90 (CKD 1–2 after 90 days: 2700 µg/ml [1579; 6972], CKD 3–5: 3980 µg/ml [1621; 7233]; (*p* = 0.395).

### Risk factors for hospital mortality

The following parameters were univariate associated with in-hospital mortality: need for KRT, AKI stage 3, need for invasive mechanical ventilation, sepsis, need for vasopressors, liver cirrhosis, active malignancy, high APACHE II or SOFA score, MAP, heart rate, myoglobin, urea, creatinine, IL-6, albumin, platelet count, pH, bicarbonate, potassium, lactate level and diuresis at admission. However, only the SOFA Score (OR: 1.111; CI: 1.004–1.228; *p* = 0.041) and need for KRT (OR: 2.163; CI: 1.061–4.407); *p* = 0.034) were independent predictive factors for hospital mortality according to multivariate logistic regression (Suppl. Table 3).

### Hospital mortality and specific kidney replacement modalities

**Fig. 3:**
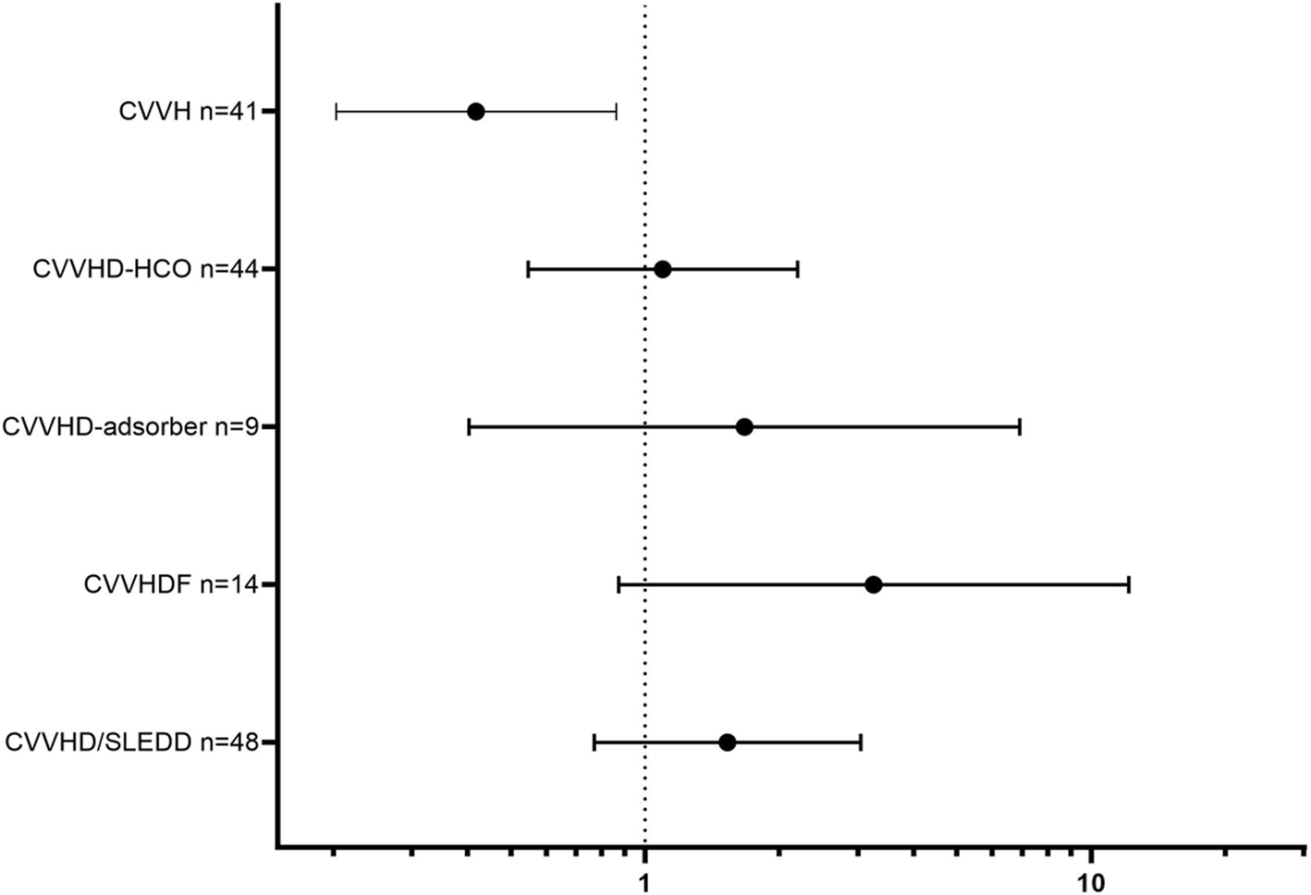
Risk for all-cause hospital mortality with the various kidney replacement modalities. CVVH continuous veno-venous hemofiltration, CVVHD-HCO continuous veno-venous hemodialysis with high cut-off filter, CVVHD-adsorber continuous veno-venous hemodialysis with “standard” high-flux filter and adsorber, CVVHDF continuous veno-venous hemodiafiltration, CVVHD continuous veno-venous hemodialysis, SLEDD sustained low efficiency daily dialysis

Survival curves of specific KRTs showed no benefit for any of the therapies (log rank *p* = 0.077) (Fig. 2D). Patient treated with CVVH seemed to have a lower hospital mortality compared to those with other specific therapies and lower compared to the predicted mortality based on APACHE II score (Table 4 and Fig. 3) [30, 31]. However, this supposed advantage for CVVH could not be confirmed after multivariate logistic regression (OR: 0.505 [0.208; 1.225]; *p* = 0.131).

**Table 4:** Hospital mortality and specific kidney replacement therapies (univariate analysis)

	**All** ***n* = 171**	**CVVH** ***n* = 41**	**CVVHD-HCO** ***n* = 44**	**CVVHD-adsorber** ***n* = 9**	**CVVHDF** ***n* = 14**	**CVVHD/SLEDD *n* = 48**	**Others** ***n* = 15**	***p***
**Hospital mortality**	95 (55.6%)	16 (39.0%)	25 (56.8%)	6 (66.7%)	11 (78.6%)	30 (62.5%)		0.015^*^
**APACHE II**	32 [7;14]	29 [24;36]	34 [25;39]	30 [26;33]	33 [27;40]	33 [26;40]		
**SOFA**	11 (7;14)	9 [5;13]	10 [7;14]	11 [9;15]	13 [9;16]	12 [7;14]		
**Predicted mortality**^a^	56.7%	45.8%	63.7%	49.5%	60.3%	60.3%		

*APACHE* Acute Physiology And Chronic Health Evaluation, *CVVH* Continuous veno-venous hemofiltration, *CVVHD* Continuous veno-venous hemodialysis, *CVVHDF* Continuous veno-venous hemodiafiltration, *HCO* High cut-off, *SLEDD* Sustained low-efficiency daily dialysis, *SOFA* Sequential organ failure assessment

^*^Chi-square test: CVVH vs. all other KRT modalities

^a^Predicted mortality based on Apache II score (reason for admission: metabolic/renal (https://clincalc.com/IcuMortality/) [31])

### Dialyzer lifetime

**Fig. 4:**
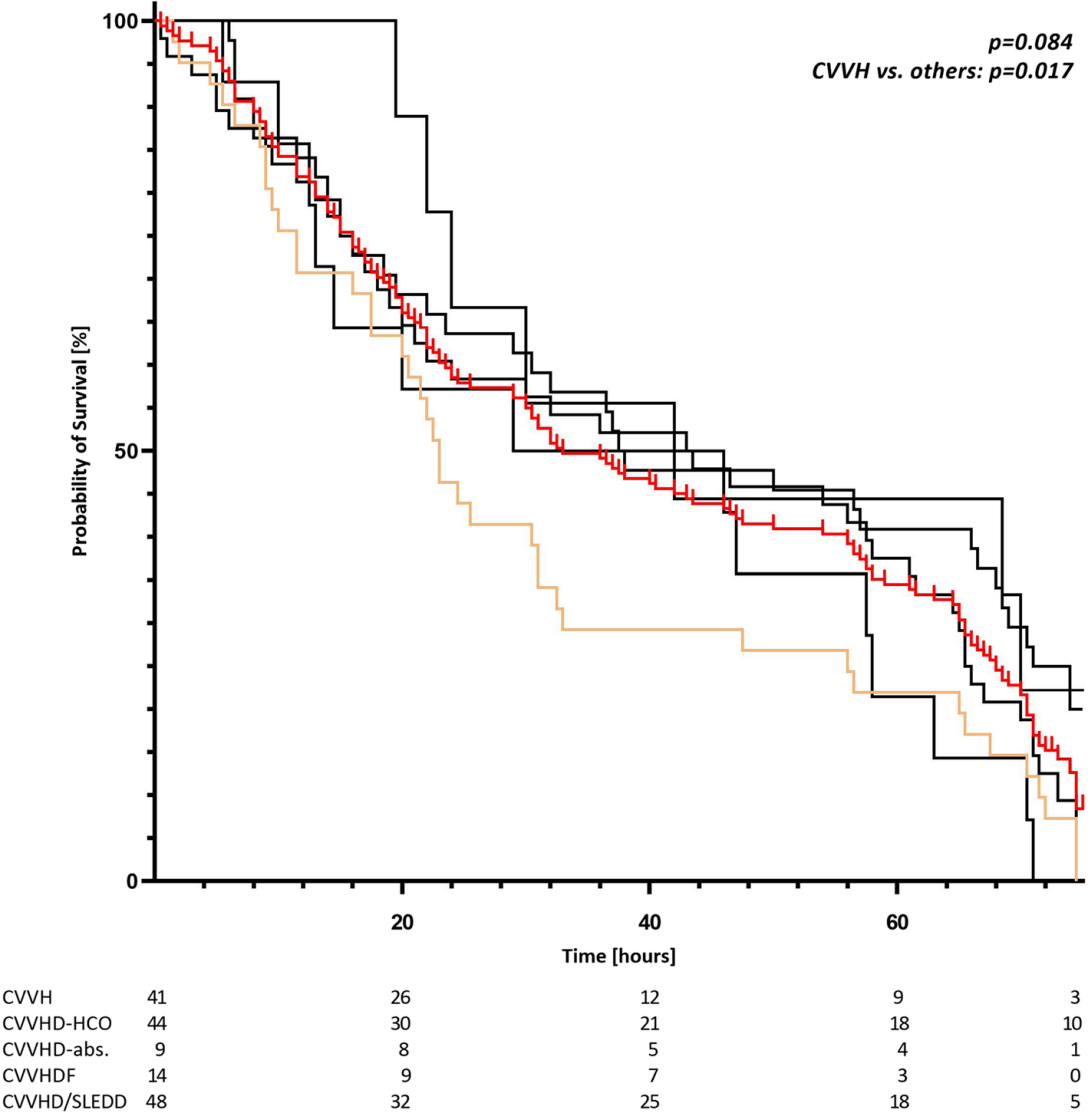
Survival of extracorporeal circuit. Kaplan–Meier curve for extracorporeal circuit survival with the various kidney replacement modalities. red line: all modalities; orange line: CVVH; black lines: CVVHD-HCO, CVVHD-adsorber, CVVHDF, CVVHD/SLEDD

Life span of the extracorporeal circuit was shorter with CVVH compared to that of all other KRT techniques (log-Rank *p* = 0.017) (Fig. 4). For other modalities significant differences could not be demonstrated in this issue.

## Discussion

The present study confirmed that AKI requiring KRT in rhabdomyolysis is associated with a high hospital mortality rate. Multivariate binary logistic regression showed that application of specific KRT therapy considering extracorporeal myoglobin elimination could not improve all-cause hospital mortality. Nevertheless, the trend for lower hospital mortality rates on CVVH may indicate that extracorporeal myoglobin elimination could have an influence on outcome. This hypothesis is supported by an older animal experiment of rhabdomyolysis, in which accelerated start of CVVH demonstrated improved morphological changes of renal mitochondria and less apoptosis [32].

Application of clinical scores can help to identify patients at increased risk for AKI and death from rhabdomyolysis [33]. However, it remains uncertain whether these patients benefit from early implementation of specific extracorporeal treatments.

Older studies define rhabdomyolysis on the basis of elevated serum creatinine kinase (CK) [34], but serum myoglobin concentrations above between 368 µg/l [22] and 3865 µg/l [23] predict development of AKI more accurate [22, 35] and might be a better diagnostic parameter in view of myoglobin as nephrotoxic substance. For this reasons, we defined rhabdomyolysis as myoglobin above 1000 µg/l in the range of published thresholds [22, 23].

In this study in 52,1% of the patients needed KRT, which is considerably more than in other studies [22, 23, 35, 36], but these did not only include critically ill patients with high disease severity and the definition of rhabdomyolysis differed in some cases [22, 23, 35, 36]. Higher myoglobin levels in this study were associated with an increased risk of hospital mortality but could not be confirmed as an independent risk factor for death and end stage kidney disease (ESKD). An impact on renal function at day 90 was also not detectable, although it is generally assumed that the severity of AKI influences the progression of CKD [37]. The number of cases and the relatively short follow-up period may have altered our results at this point.

Two studies could not confirm an association of initial CK-levels with mortality and renal outcome, but the stage of AKI was a strong predictor [38, 39]. Regarding the strong associations of CK and myoglobin levels [20, 22, 35], the prognostic value of myoglobin in predicting mortality and renal outcomes appears to be limited, but could still increase the risk for AKI [23, 35]. Furthermore, serum CK levels at the time of termination of KRT were not able to estimate clinical outcome if daily diuresis exceeds > 1 l/d [40].

The high hospital mortality rate of 55,6% in patients requiring KRT was similar to mortality rates observed in large multicenter trials [2, 3] and explained by a high APACHE II score in the KRT group [31, 41]. Only the SOFA score and the requirement of KRT were identified as independent risk factors for hospital mortality as already shown in large trials from critically ill patients [2, 3, 42]. These results suggest that general critical condition, rather than a single factor, determines hospital mortality in our study.

Best therapeutic approach for toxic myoglobin elimination is preservation of good kidney performance. Although not significant, the trend towards a better MRR with CT than with KRT in the present study may underline this fact. That is why keystones in treatment of rhabdomyolysis are optimization of kidney perfusion and the control of patient’s fluid balance to enhance urine production [43, 44]. Consequently, aggressive fluid replacement and probably supplementation with sodium bicarbonate had been recommended [6]. In contrast, in a recently published survey, patients with rhabdomyolysis did not benefit from high fluid load (> 3 ml/kg/h). Fluid even seems to be deleterious. Administration of sodium bicarbonate resulted in poor renal outcomes, too [45]. For this reason, recommendations for CT of rhabdomyolysis should be reconsidered. Extracorporeal treatment can not recommended in patients with working kidneys [43] as it provides the highest elimination capacity of myoglobin. If KRT is necessary, various modalities of KRT with different myoglobin elimination capacity are available [9, 11, 12, 14, 15, 17, 21, 32, 46–48], but good clinical data concerning outcome of patients are missing. Therefore, no recommendation for any specific method of KRT is available until now [43].

Differences in MRR between specific KRT modalities were not observed. There was only a trend towards a higher reduction rate with CVVH and CVVHD-HCO. High myoglobin reduction rate of 22 ml/min in CVVH was already observed by Amyot et al. [9]. Data from our research group already showed a better myoglobin clearance in patients treated with CVVHD-HCO compared to CVVHDF (mean difference of 5.5 (4–7) ml/min, *p* < 0.0005; (12.3 vs. 3.7 ml/min)) [12]. Another investigation could verify a relevant myoglobin elimination in CVVHD-HCO in rhabdomyolysis, too [48]. In a recently published study CVVHD-HCO combined with an adsorber had a particularly good elimination capacity [21]. However, there are differences to our study [12], which explain the lower MRR: firstly, the adsorber was not combined with an HCO filter but with a high-flux filter and secondly, the adsorbers were used for 24 h. As a result, the overall performance was probably weakened due to the rapid filter saturation in the first treatment hours. However, a MRR of 45% [35.3; 56.3] was similar to a retrospective analysis among 43 patients with a significant myoglobin reduction rate of 29% [8.8; 50] [− 29.4% (IQR: − 41.2, + 2.6%] within the first 24 h of treatment [20].

Comparison of filter lifetime in different continuous KRTs in our cohort showed shortest survival with CVVH. Maintenance of extracorporeal circuit in CVVH was ensured by systemic anticoagulation using heparin due to the need for higher blood flow rates. Filter lifetime is longer in RCA compared to systemic anticoagulation with heparin [49–51] and premature filter clotting seems more pronounced in convective procedures, because of more hemoconcentration along the filter and higher transmembrane pressures [52, 53].

This retrospective analysis found a significant association between an acidic urine and requirement of KRT. Data concerning this issue are rare. Aslan et al. could not demonstrate an association between urine pH and contrast-induced nephropathy [54], thus acidic urine in general sets not a bad prognosis. However an alkaline environment seems to stabilize ferry species within the myoglobin molecule [6]. In consequence, it reduces formation of reactive oxygen radicals and diminishes lipid peroxidation of tubular cells and mitochondrial membranes [43, 55]. It is comprehensible that acidic urine enhances kidney toxicity in rhabdomyolysis and therefore alkalization of urine seems a useful therapeutic option.

There are several limitations concerning this study. Firstly, it is a monocentric and retrospective analysis. Secondly, there may have been a beta error because of many small subgroups, particularly regarding the different KRT modalities. Thirdly, multiple statistical comparisons can randomly produce significant differences. Fourthly, complete data sets were not available for every patient. Fifthly, some important parameters in the evaluation of kidney function such as albuminuria, proteinuria or hematuria were not available. Sixthly we calculated only myoglobin reduction rates in patient´s serum. This should be distinguished from clearance rate due to an extracorporeal circuit. Differences in endogenous myoglobin production and residual renal myoglobin elimination could under- or overestimate myoglobin elimination of KRT modality.

Nevertheless, the data represent therapy and outcome of patients suffering from rhabdomyolysis from a daily setting in a large medical ICU over a period of more than 4.5 years and provides important information about this severe disease pattern. It can also be useful to generate hypotheses and to calculate adequate power for prospective randomized studies.

## Conclusions

This study emphasizes that AKI requiring KRT following rhabdomyolysis is accompanied by high mortality rate. Differences in myoglobin reduction rate between various KRTs could not be confirmed, but CVVH was associated with reduced filter lifetime compared to other KRTs, which enable myoglobin elimination, too.

### Supplementary Information

**Supplementary Material 1. **

## Data Availability

All data generated or analyzed during this study are included in this article and its supplementary material files. Further enquiries can be directed to the corresponding author.

## Abbreviations

AKI: Acute kidney injury
CKD: Chronic kidney disease
KRT: Kidney replacement therapy
CVVH: Continuous veno-venous hemofiltration
CVVHDF: Continuous veno-venous hemodiafiltration
CVVHD-HCO: Continuous veno-venous hemodialysis with high cut-off filter
SLEDD: Sustained low-efficiency daily dialysis
HCO: High cut-off
CKD: Chronic kidney disease
ICU: Intensive care unit
MRR: Myoglobin reduction rate
CT: Conservative treatment
kDa: Kilodaltons
RCA: Regional citrate anticoagulation
TTR: Total turnover rate
BMI: Body mass index
APACHE II: Acute physiology and chronic health evaluation II score
SOFA: Sequential organ failure assessment
eGFR (CKD-EPI): Estimated glomerular filtration according Chronic Kidney Disease Epidemiology Collaboration
CK: Creatin kinase
IL-6: Interleukin-6
MAP: Mean arterial pressure
ESKD: End-stage kidney disease
