# Supplementary material for: Kidney replacement and conservative therapies in rhabdomyolysis: a retrospective analysis
Source: BMC Nephrol. 2024 Mar 14;25:96. doi: 10.1186/s12882-024-03536-8 (PMC10938657; doi:10.1186/s12882-024-03536-8)
Supplement: Supplementary file 1 — Supplementary Material 1. [file 12882_2024_3536_MOESM1_ESM.docx]

| **Parameters** | **CVVH** | **CVVHD-HCO** | **CVVHD-adsorber** | **CVVHDF** |
| --- | --- | --- | --- | --- |
|  | **n=41** | **n=44** | **n=9** | **n=14** |
| **total turnover rate**  (ml/min) | 2000  [1250;2150] | 2000  [2000;2500] | 2450  [2000;3000] | 1500  [1000;2000] |
| **blood flow**  (ml/min) | 150  [120;180] | 100  [100;120] | 135  [100;160] | 150  [100;150] |
| **ultrafiltration**  (ml/h) | 0  [0;0] | 0  [0;0] | 0  [0;0] | 0  [0;0] |
| **filter lifetime**  (h) | 23  [11; 56] | 38  [15;72] | 42  [23;71] | 46  14;61] |

**Supplemental table 1: Parameters of kidney replacement therapy**

*CVVH continuous veno-venous hemofiltration, CVVHD continuous veno-venous hemodialysis, HCO high cut-off, CVVHDF continuous ven-ovenous hemodiafiltration*

| **Myoglobin measurement (time)** | **all** | **KRT** | **CT** |
| --- | --- | --- | --- |
| **At inclusion** | 328 | 171 | 157 |
| **Morning after inclusion** | 270 | 141 | 136 |
| **After 24-hours (day 2)** | 213 | 125* | 90 |
| **After 48-hours (day 3)** | 138 | 87 | 46 |
| **After 72-hours (day 4)** | 105 | 77 | 23 |

**Supplemental table 2: Number of cases**

| **Parameters** | **CVVH** | **CVVHD-HCO** | **CVVHD-adsorber** | **CVVHDF** |
| --- | --- | --- | --- | --- |
|  | **n=41** | **n=44** | **n=9** | **n=14** |
| **total turnover rate**  (ml/min) | 2000  [1250;2150] | 2000  [2000;2500] | 2450  [2000;3000] | 1500  [1000;2000] |
| **blood flow**  (ml/min) | 150  [120;180] | 100  [100;120] | 135  [100;160] | 150  [100;150] |
| **ultrafiltration**  (ml/h) | 0  [0;0] | 0  [0;0] | 0  [0;0] | 0  [0;0] |
| **filter lifetime**  (h) | 23  [11; 56] | 38  [15;72] | 42  [23;71] | 46  14;61] |

**Discrepancy to n=118 (table 3) included patients to KRT specific myoglobin reduction rate analysis explained by missing data at “morning after inclusion”*

**Supplemental table 3: Patient characteristics and hospital mortality (multivariate analysis), n=325**

| **Parameters** | **OR** | **CI** | ***p*** |
| --- | --- | --- | --- |
| **KRT vs. CT** | 2.163 | 1.061-4.407 | **0.034** |
| **SOFA** | 1.111 | 1.004-1.228 | **0.041** |
| **APACHE II** | 1.045 | 0.997-1.096 | 0.065 |
| **MAP** | 0.998 | 0.985-1.012 | 0.788 |
| **HR** | 0.998 | 0.986-1.010 | 0.730 |
| **myoglobin** | 1.000 | 1.000-1.000 | 0.345 |
| **urea** | 1.018 | 0.986-1.052 | 0.277 |
| **creatinine** | 0.997 | 0.995-1.000 | 0.062 |
| **pH** | 0.578 | 0.026-13.013 | 0.730 |
| **HCO_3_^-^** | 1.013 | 0.947-1.082 | 0.711 |
| **lactate** | 1.058 | 0.976-1.148 | 0.172 |
| **potassium** | 1.281 | 0.905-1.815 | 0.163 |
| **mechanical ventilation** | 1.226 | 0.547-2.748 | 0.621 |
| **vasopressors** | 1.513 | 0.719-3.181 | 0.275 |
| **sepsis** | 1.101 | 0.581-2.088 | 0.768 |
| **AKI 3** | 1.224 | 0.602-2.489 | 0.576 |
| **liver cirrhosis** | 2.711 | 0.801-9.175 | 0.109 |
| **active malignancy** | 3.157 | 0.865-11.517 | 0.082 |

*AKI acute kidney injury, APACHE Acute Physiology And Chronic Health Evaluation****,*** *CT conservative treatment, HR heart rate, KRT kidney replacement therapy, MAP mean arterial pressure, OR odds ratio, CI confidence interval (95%), SOFA sequential organ failure assessment*
